# Cutaneous *Mycobacterium szulgai* infection in a patient with Cushing's syndrome: a case report and literature review

**DOI:** 10.1186/s12879-023-08253-5

**Published:** 2023-04-26

**Authors:** Haiyan Ye, Vanessa C. Harris, Kelvin Hei-Yeung Chiu, Shuang Chen, Fanfan Xing, Linlin Sun, Chaowen Deng, Jin Yang, Jasper Fuk-Woo Chan, Kwok-Yung Yuen

**Affiliations:** 1 Department of Infectious Disease and Microbiology, The University of Hong Kong-Shenzhen Hospital, Shenzhen, Guangdong China; 2 Department of Internal Medicine, Division of Infectious Diseases and Department of Global Health, Amsterdam University Medical Centers, University of Amsterdam, Amsterdam, Netherlands; 3 Department of Global Health, Amsterdam Institute for Global Health and Development, Amsterdam University Medical Centers, University of Amsterdam, Amsterdam, Netherlands; 4 Amsterdam Institute of Infection and Immunity, Infectious Diseases, Amsterdam, Netherlands; 5 Department of Microbiology, Queen Mary Hospital, Hong Kong Special Administrative Region, Pokfulam, China; 6 Department of Pathology, The University of Hong Kong-Shenzhen Hospital, Shenzhen, Guangdong China; 7 State Key Laboratory of Emerging Infectious DiseasesDepartment of Microbiology, School of Clinical MedicineFaculty of Medicine, Carol Yu Centre for InfectionLi Ka ShingThe University of Hong KongHong Kong Special Administrative Region, Pokfulam, China

**Keywords:** *Mycobacterium szulgai*, Cushing’s syndrome, Hypercortisolism, Cutaneous infection, Case report

## Abstract

**Background:**

Opportunistic infection is an under-recognized complication of Cushing’s syndrome, with infection due to atypical mycobacterium rarely reported. *Mycobacterium szulgai* commonly presents as pulmonary infection, with cutaneous infection seldom reported in the literature.

**Case Presentation:**

48-year-old man with a newly-diagnosed Cushing’s syndrome secondary to adrenal adenoma presented with a subcutaneous mass on the dorsum of his right hand, was diagnosed with cutaneous *Mycobacterium szulgai* infection. The most likely source of the infection was through minor unnoticed trauma and inoculation from a foreign body. The patient’s Cushing’s syndrome, high serum cortisol levels and secondary immune suppression facilitated mycobacterial replication and infection. The patient was successfully treated with adrenalectomy, surgical debridement of cutaneous lesion, and a combination of rifampicin, levofloxacin, clarithromycin, and ethambutol for 6 months. There were no signs of relapse one year after cessation of anti-mycobacterial treatment. A literature review on cutaneous *M. szulgai* infection to further characterize the clinical characteristics of this condition, identified 17 cases of cutaneous *M. szulgai* infection in the English literature. Cutaneous *M. szulgai* infections with subsequent disease dissemination are commonly reported in immunocompromised hosts (10/17, 58.8%), as well as in immunocompetent patients with a history of breached skin integrity, such as invasive medical procedures or trauma. The right upper extremity is the most commonly involved site. Cutaneous *M. szulgai* infection is well controlled with a combination of anti-mycobacterial therapy and surgical debridement. Disseminated infections required a longer duration of therapy than localized cutaneous infections. Surgical debridement may shorten the duration of antibiotics.

**Conclusions:**

Cutaneous *M. szulgai* infection is a rare complication of adrenal Cushing’s syndrome. Further studies are needed to provide evidence-based guidelines on the best combination of anti-mycobacterial and surgical therapy for managing this rare infective complication.

## Introduction

Cushing's syndrome is due to prolonged exposure to excess glucocorticoids, and common causes include exogenous steroid use, adrenal or pituitary ACTH-secreting tumors or ectopic ACTH syndrome. Opportunistic infection is an under-recognized complication of Cushing’s syndrome. Common opportunistic infections reported in the literature include *Cryptococcus, Aspergillus*, *Nocardia,* and *Pneumocystis jirovecii* infection [1]; atypical mycobacterial infections have seldomly been reported in patients with Cushing’s syndrome [2].

*M. szulgai* was first described in 1972 [3]. It is a rare pathogen and accounts for less than 0.5% of all nontuberculous mycobacterial (NTM) infections [4]. *M. szulgai* infections present with a variety of clinical manifestations, ranging from predominantly pulmonary to rarely cutaneous infection [5–8]. Here we report the first case of cutaneous *Mycobacterium szulgai* infection in a patient with ACTH-independent Cushing’s syndrome, and summarize the literature on cutaneous *M. szulgai* infection.

### Case presentation

In November 2017, a 48-year-old man was admitted to our hospital in Shenzhen, China. He presented with a mass at the dorsum of his right hand without systemic symptoms. Four months prior to the presentation, the patient had noticed the development of subcutaneous mass at the dorsum of the right wrist following a routine cleaning of his tropical fish tank. He noted that during the cleaning he had injured his right wrist but did not notice any external wounds. In the course of the following 4 months the lesion gradually increased in size. The patient first presented to an outside hospital where Magnetic Resonance Imaging (MRI) revealed a 7.0 cm × 6.0 cm cystic mass at the dorsum of the right hand. Subsequent aspiration and drainage of the cyst yielded pus, but bacterial culture was not performed. The patient was empirically treated with 1-month course of oral cephalosporin. However, despite drainage, the cystic mass recurred and the patient was referred to our hospital for further management.

Further history revealed a 10 kg weight gain in the preceding 5 years. He had no constitutional symptoms and no personal and contact history of pulmonary tuberculosis.

The patient had a 5-year history of poorly controlled hypertension requiring amlodipine, furosemide, and spironolactone. He also reported long-standing right ulnar nerve palsy following an accident in 1996. He was a non-smoker and non-drinker. He had no history of recreational drug use and no recent travel history. He denied other animal exposure besides fish tank cleaning. He denied the use of over-the-counter medications, including oral steroids or other immunosuppressive agents.

**Fig. 1:**
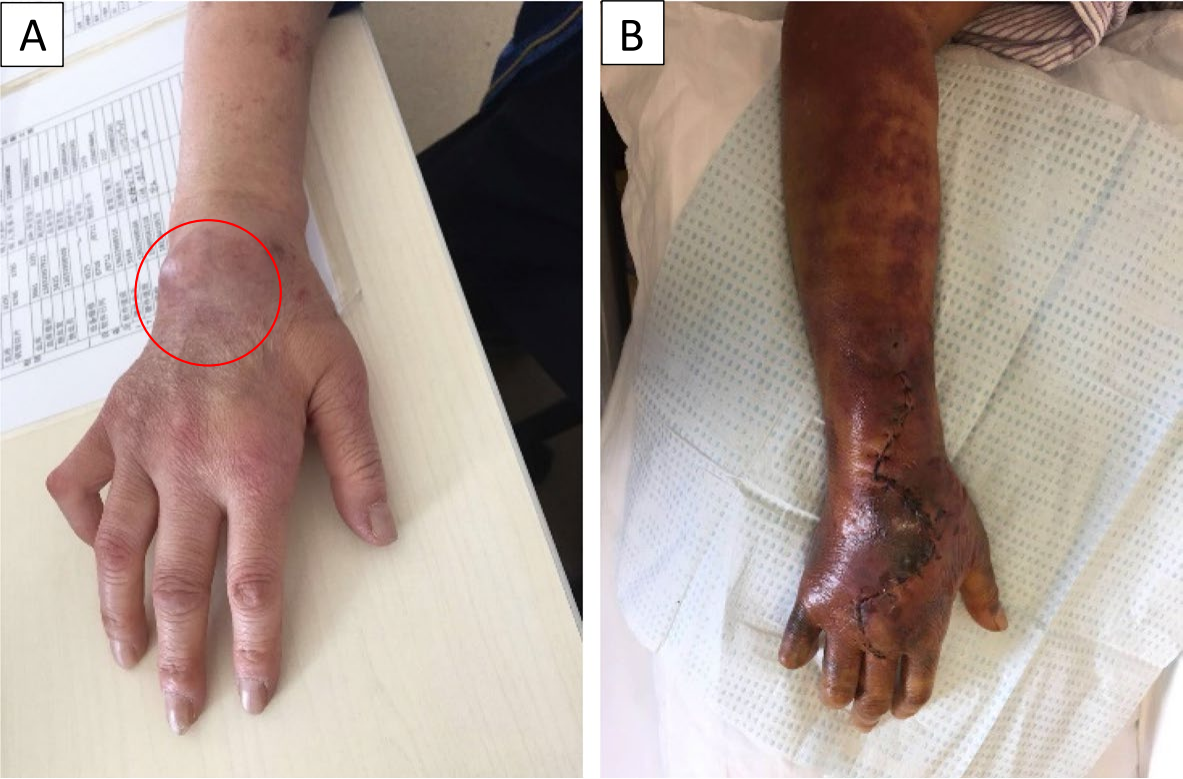
Clinical photos of the patient's right upper limb. **A** Subcutaneous mass located at the right dorsal wrist on the day before operation; **B** Swelling and ecchymoses after 10 days of incision and debridement

On presentation to our hospital, the mass over the dorsum of the right wrist was 7.0 cm × 6.0 cm in size (Fig. 1A). The mass was surgically debrided and tenosynovitis with extensive inflammatory granulation tissue and pus was noted at the time of the operation. Postoperative healing of the surgical wound was poor despite adequate debridement, and the possibility of atypical infection was considered.

Ten days after incision and debridement, physical examination of the patient revealed a moon-shaped face with facial plethora, prominent supraclavicular fat pads, truncal obesity, and wide purplish abdominal striae. The right hand was edematous with multiple ecchymoses and serous exudates (Fig. 1B), and without evidence of erythema, warmth, and tenderness. There was no palpable trochlear or axillary lymphadenopathy. His body mass index was 21.5 kg/m^2^.

**Fig. 2:**
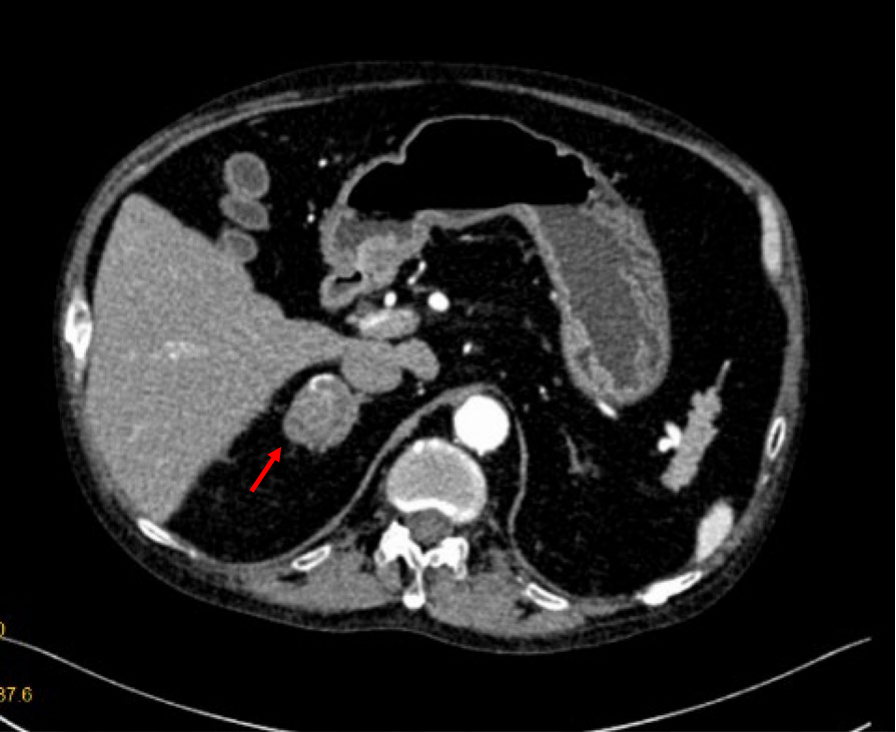
Computed tomography scan of the abdomen showed a 3 × 3 cm right adrenal mass as indicated by the red arrow

Laboratory examination showed an elevated white blood cell count (11,870 cells/μL), neutrophil count (10,270 cells/μL), and lymphocyte count (740 cells/μL), but normal platelet count (342,000 cells/μL), erythrocyte sedimentation rate (32 mm/h), C-reactive protein (8.7 mg/L), fasting serum glucose level (6.4 mmol/L) and sodium level (143 mmol/L). The potassium level was low (2.44 mmol/L). Further laboratory testing revealed a high morning cortisol level (35.9 μg/dL) and low ACTH level (< 1.00 pg/mL). A computed tomography scan of the abdomen showed an enlarged right adrenal gland (Fig. 2). The diagnosis of ACTH-independent Cushing’s syndrome secondary to a right adrenal adenoma was made.

**Fig. 3:**
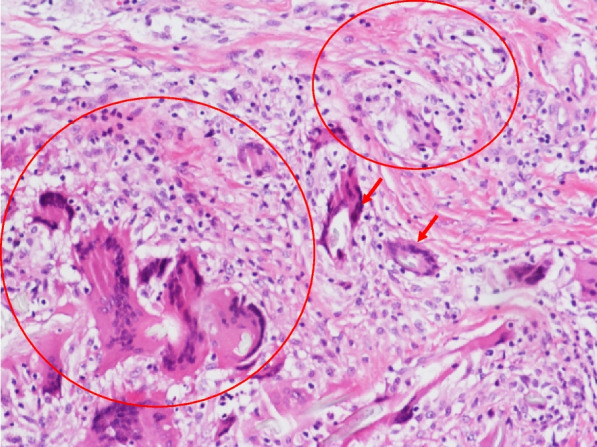
Histology of the excised mass at right hand dorsum showed non-caseating granulomas (circles) surrounding isolated foreign bodies (arrows) with nonspecific chronic inflammation (Hematoxylin and eosin stain: 10 × 40)

Histology of the excised cystic mass showed non-caseating granulomas surrounding isolated foreign bodies with nonspecific chronic inflammation (Fig. 3). Ziehl–Neelsen, Periodic Acid-Schiff, and methenamine silver stains were negative. The operative tissue was negative for AFB smear and TB DNA. The operative tissue was sent to the Tuberculosis Reference Center of Shenzhen, with *M. szulgai* grown from Löwenstein-Jensen medium following 14 days of incubation in BACTEC MGIT 960 system. Susceptibility testing of the strain was not performed according to protocol.

The diagnosis of cutaneous *M. szulgai* infection was based on a positive culture in combination with histopathology demonstrating granuloma formation surrounding foreign bodies without caseation. The right adrenal adenoma was excised and it was subsequently followed by replacement and tapering doses of steroid. Patient was concomitantly started on anti-mycobacterial treatment, with a combination of rifampin 600 mg once daily, clarithromycin 500 mg twice daily, levofloxacin 500 mg once daily and ethambutol 1 g once daily for 6 months. The patient tolerated anti-mycobacterial treatment well with no notable side-effects. There was significant improvement of clinical symptoms following the initiation of treatment. There were no signs of relapse one year after cessation of anti-mycobacterial treatment.

### Literature review

*M. szulgai* infection is a rare non-tuberculous mycobacterium (NTM) that can cause human infection. Pulmonary infection is the the most common manifestation of *M. szulgai* infection. Cutaneous infection due to *M. szulgai* is rarely reported. Literature review was performed using the search terms ‘cutaneous infection, soft tissue infection’ and ‘*Mycobacterium szulgai’* in PubMed. Only articles in English published before December 31, 2021, and articles that were accessible to the authors were included in the review.

## Results

### Patient characteristics

Seventeen cases of cutaneous *M. szulgai* infection reported from 1985 to 2021 were identified in the search. Clinical details are summarized in Table 1. The majority of the patients were male (11 out of 17 patients (64.7%)), with ages ranging from 4 to 77 years old (median age 51 years old) (Table 2). Ten patients (58.8%) were immunocompromised due to underlying hematological malignancy, acquired immunodeficiency syndrome (AIDS), solid organ transplantation, or use of immunosuppressive agents. Among the 7 immunocompetent patients, the predisposing factors reported include injury or implantation of foreign body, and surgical site infection, while two patients reported no apparent predisposing factors.

**Table 1:** Characteristics of the seventeen patients with cutaneous *M. szulgai* infection

Year/County	Age (years)	Sex	Comorbidity	Exposure history	Cutaneous involvement	Other organs involvement	Susceptibility test	Treatment	Duration (months)	Outcome	Ref
1985/USA	51	Male	Steroid use	Unknown	Right ankle and elbow, left knee, back	Osteomyelitis of the right ankle and fibula, pulmonary	Nil	INH, RIF, EMB	24	Cure	[9]
2000/Japan	41	Female	SLE	Unknown	Right forearm	Nil	S: INH, RIF, EMB	Debridement, CL	3	Cure	[10]
2002/Sweden	4	Male	Post-BMT	Unknown	Right big toe	Right inguinal lymphadenopathy	S: AMI, STM CIP, EMB, CL, RIF R: INH, PTO	RIF, EMB	9	Cure	[11]
2003/UK	27	Male	Anemia and lymphopenia	Unknown	Left hand to the arm, thighs	Left axillary lymphadenopathy	S: RIF, EMB, CL, AMI, CIP	INH, EMB, CL	2	Cure	[12]
2004/Germany	36	Male	AIDS	Tropical fish	Left forearm, right hand,	Osteomyelitis of the third right metacarpal joint	Nil	EMB, CL, CIP	12	Cure	[13]
2007/UK	26	Male	Unknown	Unknown	Face, arms, hands, trunk and legs	Nil	Nil	INH, RIF, EMB, PZA	2	Recurrence	[14]
2008/USA	66	Female	CLL	Unknown	Right hand, elbow, forearm, left thigh	Multifocal osteomyelitis	S: INH, RIF, EMB, AMI, CIP, CL	INH, RIF, EMB	24	Cure	[15]
2011/Japan	59	Male	HBV carrier	Unknown	Bilateral fingers and feet, left upper arm	Pulmonary	Nil	INH, RIF, PZA, STM	2	Cure	[16]
2012/UK	59	Female	Unknown	Tropical fish	Right index finger	Nil	Nil	Debridement, RIF, EMB, CL,	1.5	Cure	[17]
2015/India	46	Female	Laparoscopic appendectomy in 1998	Unknown	Abdominal wall	Nil	S: LIN, RIF, AMI, CIP, LEV, CL, EMB; R: INH	RIF, EMB, CL	4	Cure	[18]
2016/Japan	59	Male	MDS	Unknown	Left chest wall	Mediastinal lymphadenopathy	Nil	LEV, AZM, EMB	12	Cure	[19]
2017/Venezuela	36	Female	Augmentation mammoplasty	Unknown	Right breast	Nil	S: INH, RIF, EMB R: STM	Debridement, INH, RIF, EMB	8	Cure	[20]
2017/USA	66	Male	Renal transplant	Oyster	Right index finger	Nil	Nil	Debridement, EMB, MOX, AZM	4	Cure	[21]
2017/UK	53	Female	Unknown	Rose thorn injury	Right index finger	Nil	Nil	Debridement, INH, RIF, EMB, PZA	9	Cure	[22]
2019/UK	64	Male	Steroid use	Mississippi mud turtle	Right wrist	Nil	Nil	Debridement, RIF, EMB, LIN,	9	Cure	[23]
2019/China	36	Male	Heavy smoker	Sickle injury 20 years ago	Left hand, right ear, left elbow	Nil	S: EMB, CL, AMI, RIF, CIP	RIF, EMB, CL	6	Cure	[24]
2021/USA	77	Male	Steroid use	Unknown	Right hand, right wrist	Nil	Nil	Debridement, RIF, CIP, CL	3.5*	Cure	[25]

^*^3 months and half months of 3 drugs, 1 month of CIP and CL

*AIDS* Acquired immunodeficiency syndrome, *MDS* Myelodysplastic syndrome, *BMT* Bone marrow transplant, *CLL* Chronic lymphocytic leukemia, *HBV* Hepatitis B virus, *SLE* Systemic lupus erythematosus, *INH* Isoniazid, *RIF* Rifampin, *EMB* Ethambutol, *PZA* Pyrazinamide, *CL* Clarithromycin, *AMI* Amikacin, *STM* Streptomycin, *CIP* Ciprofloxacin, *LIN* Linezolid, *AZM* Azithromycin, *LEV* Levofloxacin, *MOX* Moxifloxacin, *PTO* Protionamide

**Table 2:** Demographic data and patients’ characteristics

Characteristics	*N* = 17
Age (years)	4 – 77 (Median 55)
Gender	
Female	6 (35.3%)
Male	11 (64.7%)
Comorbidity	
Immunocompromised patient	10 (58.8%)
Immunocompetent patient	7 (41.2%)
Contact history	
Tropical fish	2 (11.8%)
Oyster	1 (5.9%)
Mississippi mud turtle	1 (5.9%)
Rose thorn	1 (5.9%)
Sickle	1 (5.9%)
Site of cutaneous infection	
Upper extremity	12 (70.6%)
Lower extremity	6 (35.3%)
Breast	1 (5.9%)
Chest wall	2 (11.8%)
Abdominal wall	1 (5.9%)
External ear canal	1(5.9%)

### Organ involvement

Disseminated infection (defined as involvement of more than one anatomical site or organ) was reported in 10 out of 17 patients (58.8%) in our study. Lymphadenopathy was also reported (3/10, 30.0%). Other infected organ systems included pulmonary (2/10, 20%) and musculoskeletal systems (3/10, 30.0%). Common sites of involvement included upper extremities (12/17, 70.6%), lower extremities (6/17, 35.3%), chest wall (2/17, 11.8%), with a single case involving abdominal wall (1/17, 5.9%), breast (1/17, 5.9%), and external ear canal (1/17, 5.9%).

### Susceptibility test and treatment

Susceptibility testing for *M. szulgai* was included in 7 reported cases, with strains susceptible to most anti-mycobacterial agents, with the exception of two strains that were resistant to isoniazid, one strain resistant to streptomycin, and another resistant to protionamide. There are no standard recommendations for *M. szulgai* infection treatment to date. Most patients received combinations of 2 to 4 antimycobacterial drugs. The treatment duration reported varied widely, ranging from 6 weeks to 24 months. Patients with disseminated infection tend to receive longer duration of anti-mycobacterial drugs when compared with those with localized cutaneous infection. Seven patients received surgical debridement, with subsequent short course of anti-mycobacterial drugs (1.5 to 4 months). None of these patients progressed to disseminated infection. The prognosis of cutaneous *M. szulgai* infection is good, with only one patient who had disseminated infection developed disease relapse after cessation of a 2 months course of anti-mycobacterial treatment.

## Discussion and conclusions

*M. szulgai* is a slow-growing mycobacterium that was first described in 1972, and it was named after the Polish microbiologist Teofil Szulga [3]. *M. szulgai* has been recovered from numerous environmental water sources, including water from hospital taps, ice machines, fish tanks, and swimming pools [26]. *M. szulgai* produces smooth or rough pigmented colonies after 2–4 weeks of incubation. It is scotochromogenic at 37 °C but photochromogenic at 25 °C. In contrast to other NTM, which are often interpreted as environmental contaminants, the identification of *M. szulgai* should be considered as a significant human pathogen [27]. Pulmonary infection is the most common localized manifestation of *M. szulgai*. Extrapulmonary manifestations include cutaneous infection, cervical lymphadenitis, osteomyelitis, tenosynovitis, bursitis, keratitis, and disseminated infections associated with AIDS or other immunocompromised conditions [17, 28–32].

Cutaneous *M. szulgai* infections are most commonly reported in immunocompromised hosts and patients with history of exposure that leads to the breach of skin integrity, such as invasive medical procedures or trauma. Exposures identified in this case review included environmental cutaneous traumas such as from a rose thorn or metal sickle to aquatic animal exposure (tropical fish, oyster, and Mississippi mud turtle), with contact with tropical fish being most commonly implicated in cutaneous *M. szulgai* infection [26]. Risk factors commonly reported for pulmonary *M. szulgai* infection, such as alcoholism, smoking, chronic obstructive pulmonary disease, and history of pulmonary tuberculosis, were not identified in these case reports of cutaneous infection [33]. Among the patients with cutaneous *M. szulgai* infection in our study, the right upper limb was the most common site of involvement. This is likely because most patients were right-handed and they acquired *M. szulgai* from environmental sources through the breach of skin barrier. Our patient had loss of sensation over the ulnar aspect of his wrist and hand due to ulnar nerve palsy for many years. It is conceivable that this could have predisposed him to minor injuries.

*M szulgai* is commonly isolated from water sources and can result in surgical site infections. Two patients in our literature review had cutaneous *M. szulgai* infection that were likely iatrogenic in origin after breast implantation and appendicectomy. Holmes et al.reported a cluster of cases of *M. szulgai* keratitis following laser-assisted in situ keratomileusis. Subsequent outbreak investigation cultured *M. szulgai* from the corneal scrapings as well as the drain of the ice machine used for chilling of syringes for intraoperative lavage [28]. Cutaneous *M. szulgai* infections are more likely to be primary infections and not secondary to disseminated disease.

There are no standard guidelines on the drug treatment of *M. szulgai* infection to date. Reports describe variable susceptibility to anti-mycobacterial drugs. Case reports reviewed in this study revealed that most strains were susceptible to antimycobacterial agents with the exception of two strains that were resistant to isoniazid [34, 35]. Thus, most studies employed regimens containing rifamycin, ethambutol, and clarithromycin with or without a fluoroquinolone, which appears to be associated with a favorable outcome [5]. Literature reported a longer duration (over 6 months) of anti-mycobacterial treatment for disseminated infections, and a short course of treatment (less than 4 months) for localized cutaneous infection. Adjunctive surgical debridement to reduce bacterial load and duration of anti-mycobacterial treatment was a commonly-used treatment strategy. In our case, the *M. szulgai* infection and Cushing’s syndrome resolved following treatment with anti-mycobacterial agents in conjunction with adrenalectomy and surgical debridement of the skin lesion. A multidisciplinary management approach involving surgeons, radiologists, endocrinologists, and infectious disease physicians supported treatment in this complicated infection.

Cushing’s syndrome is associated with an increased risk of opportunistic infections. Approximately 10 percent of cases of overt Cushing's syndrome are due to adrenal adenomas. Patients with exogenous Cushing’s syndrome are more likely to be predisposed to opportunistic infections than patients with endogenous Cushing’s syndrome because of higher levels of circulating glucocorticoids and immunosuppression. Previous meta-analysis has shown that common opportunistic infections associated with Cushing’s syndrome include *Cryptococcus, Aspergillus*, *Nocardia,* and *Pneumocystis jirovecii* infection [1]. *M. szulgai* infection is uncommon in Cushing’s syndrome, with only one case of cutaneous *M. szulgai* infection reported thus far [2]. To the best of our knowledge, our case is the first *M. szulgai* infection reported in a patient with Cushing’s syndrome in China. Clinicians should be aware of the possibility of NTM infection in patients with Cushing’s syndrome.

In conclusion, cutaneous *M. szulgai* infection is a rare complication of adrenal Cushing’s syndrome. Further studies are needed to provide evidence-based guidelines on the best combination of anti-mycobacterial and surgical therapy for managing this rare infective complication.

## Data Availability

The data are available from the corresponding author upon reasonable request.

## Abbreviations

ACTH: Adrenocorticotropic hormone
AIDS: Acquired immunodeficiency syndrome
M. szulgai: Mycobacterium szulgai
MRI: Magnetic Resonance Imaging
NTM: Non-tuberculous mycobacterium
